# Top caregiver concerns in Rett syndrome and related disorders: data from the US natural history study

**DOI:** 10.1186/s11689-023-09502-z

**Published:** 2023-10-13

**Authors:** Jeffrey L. Neul, Timothy A. Benke, Eric D. Marsh, Bernhard Suter, Lori Silveira, Cary Fu, Sarika U. Peters, Alan K. Percy, for the Rett syndrome Natural History Study Group, Steven A. Skinner, Peter T. Heydemann, Robin C. Ryther, Richard H. Haas, David N. Lieberman, Art A. Beisang, Timothy Feyma, Shannon M. Standridge

**Affiliations:** 1 Department of Pediatrics, Vanderbilt Kennedy Center, Vanderbilt University Medical Center, Nashville, TN USA; 2 University of Colorado School of Medicine/Children’s Hospital Colorado, Aurora, CO USA; 3 Children’s Hospital of Philadelphia, University of Pennsylvania Perelman School of Medicine, Philadelphia, PA USA; 4 Baylor College of Medicine, Houston, TX USA; 5 University of Alabama at Birmingham, Birmingham, AL USA

**Keywords:** Rett syndrome, CDKL5, FOXG1, *MECP2* duplication, Neurodevelopmental disorders, Caregiver concerns

## Abstract

**Objective:**

Recent advances in the understanding of neurodevelopmental disorders such as Rett syndrome (RTT) have enabled the discovery of novel therapeutic approaches that require formal clinical evaluation of efficacy. Clinical trial success depends on outcome measures that assess clinical features that are most impactful for affected individuals. To determine the top concerns in RTT and RTT-related disorders we asked caregivers to list the top caregiver concerns to guide the development and selection of appropriate clinical trial outcome measures for these disorders.

**Methods:**

Caregivers of participants enrolled in the US Natural History Study of RTT and RTT-related disorders (*n* = 925) were asked to identify the top 3 concerning problems impacting the affected participant. We generated a weighted list of top caregiver concerns for each of the diagnostic categories and compared results between the disorders. Further, for classic RTT, caregiver concerns were analyzed by age, clinical severity, and common RTT-causing mutations in *MECP2*.

**Results:**

The top caregiver concerns for classic RTT were effective communication, seizures, walking/balance issues, lack of hand use, and constipation. The frequency of the top caregiver concerns for classic RTT varied by age, clinical severity, and specific mutations, consistent with known variation in the frequency of clinical features across these domains. Caregivers of participants with increased seizure severity often ranked seizures as the first concern, whereas caregivers of participants without active seizures often ranked hand use or communication as the top concern. Comparison across disorders found commonalities in the top caregiver concerns between classic RTT, atypical RTT, *MECP2* duplication syndrome, CDKL5 deficiency disorder, and FOXG1 syndrome; however, distinct differences in caregiver concerns between these disorders are consistent with the relative prevalence and impact of specific clinical features.

**Conclusion:**

The top caregiver concerns for individuals with RTT and RTT-related disorders reflect the impact of the primary clinical symptoms of these disorders. This work is critical in the development of meaningful therapies, as optimal therapy should address these concerns. Further, outcome measures to be utilized in clinical trials should assess these clinical issues identified as most concerning by caregivers.

**Supplementary Information:**

The online version contains supplementary material available at 10.1186/s11689-023-09502-z.

## Introduction

Rett syndrome (RTT) is a severe neurodevelopmental disorder (NDD) that predominantly, but not exclusively [1], affects girls and women and is characterized by regression with loss of acquired spoken language and volitional hand use, disrupted or absent ambulation, and repetitive hand movements [2]. Affected individuals are impacted by a variety of additional clinical problems such as seizures, autonomic and breathing abnormalities, growth failure, scoliosis, and gastrointestinal and nutritional symptoms [3–5]. RTT is caused, in most cases, by loss of function mutations in the X-linked gene *methyl-CpG-binding protein 2* (*MECP2*) [6, 7]. Animal models of RTT [8–11] provide insight into underlying pathophysiology and facilitate the development of potential therapeutic interventions with the potential to significantly benefit affected people or even modify the disease course [12]. This has led to the initiation of clinical trials in RTT [13–15], including recent FDA approval of trofinetide for RTT [16] and the proposal for additional trials to evaluate novel treatment approaches including gene therapy. 

Critical to successful clinical therapeutic development is detailed knowledge about the disease course, clinical features, and availability of outcome measures that are both psychometrically valid and assess critical clinical domains. Extensive information on the spectrum of clinical features and disease progression in RTT has been acquired from the US Natural History Study (NHS) of RTT and RTT-related disorders, which enrolled people with RTT and disorders with clinical and genetic relationships to RTT: *MECP2* duplication syndrome (MDS); CDKL5 deficiency disorder (CDD); and FOXG1 syndrome (FS). These other disorders have been considered RTT-related due to observed clinical similarities between the disorders, and previously CDD and FS were considered to be forms of “atypical RTT” [2]; however, they are now recognized as distinct clinical disorders [17, 18]. The NHS information, combined with other large disease databases [19], has been instrumental in establishing clinical trial readiness through the development of distinct outcome measures [20–23], identifying putative biomarkers [24–26], and supporting clinical trials in CDKL5 deficiency disorder (CDD) [18, 27]. While these efforts are essential for clinical trials, knowing which clinical issues and problems are most concerning and impactful for affected individuals is necessary to develop therapies that meaningfully address these concerns. Outcome measures are needed that assess those impactful problems relevant to affected individuals and their caregivers [28]. The US Food and Drug Administration (FDA) recognizes the importance of receiving meaningful input from affected individuals on the most important concepts (disease symptoms and impact) to inform the development of outcome measures [29] and has provided guidance on methods to obtain this information from affected people and other key stakeholders [30]. A challenge in severe NDDs such as RTT is that affected people have markedly impaired communication precluding direct ascertainment from the affected individuals. Caregiver reports of meaningful issues and concerns provide a way to develop this understanding and have been utilized in other severe NDDs [31–33]. The FDA has recognized that such caregiver-reported information is needed for affected individuals with cognitive limitations [30].

To identify the top caregiver concerns in RTT and RTT-related disorders, we utilized the US NHS data obtained from 2014 to 2021. During this period, parents or caregivers were asked at every study visit to select the top 3 concerns for the affected individual under their care. Our objective was to identify the top caregiver concerns in Classic RTT and evaluate variation in the top caregiver concerns in relation to age, *MECP2* mutation, overall clinical severity, and specific clinical features such as seizures, hand use, ambulation, and spoken language. We hypothesized that caregiver concerns would align with common clinical features observed in Classic RTT and vary based on age-related and overall severity-associated frequency and severity of clinical problems, but we also considered that the top concerns identified by caregivers may deviate from clinical problems commonly acknowledged by clinicians. Furthermore, we sought to characterize the top caregiver concerns in Atypical RTT, MDS, CDD, and FS, and compare the concerns across the disorders. We hypothesized, based on clinical similarities shared across these disorders, that there would be a degree of consistency in the top caregiver concerns between these disorders, but differences in top caregiver concerns between these disorders would exist and align with known clinical differences between these distinct disorders. The work described here provides critical information on the top caregiver concerns in these disorders, identifying similarities and important differences, and represents important information identifying clinical issues that new therapies should target and can help guide the development and selection of outcome measures that assess most meaningful concerns. Despite relatively similar functional levels of individuals with each disorder, differences also emerge among the RTT-related disorders that should be considered. Thus, these top concerns do not simply reflect the presence of intellectual disability but also reflect the specific phenotypes of the respective disorders.

## Methods

### Participant information

The Rett syndrome and RTT-related Disorders Natural History Study (NHS) longitudinally collected caregiver-provided historical and clinically assessed information from participants with RTT (classic or atypical), people who had pathogenic variants in *MECP2* but did not meet clinical criteria for RTT, and people with RTT-related disorders (MDS, CDD, FS) from 2006 to 2021 through three rounds of funding from the NIH (HD061222). Participants were recruited from RTT clinics and through patient advocacy groups (PAGs), including identifying PAG-associated caregivers from underrepresented groups to increase the diversity of participants. Participants were assessed in a structured in-person clinical research visit (lasting ~ 1–2 h), which occurred longitudinally at pre-defined intervals based on age of enrollment, ranging from yearly to every other year. In-person evaluations utilizing structured research forms including caregiver-completed history and assessment forms and questionnaires, clinical histories, structured clinical exams, and clinician-completed rating scales. Clinical assessment and rating scales were conducted by physician investigators who were trained on the conduct of the study and the completion of the forms via in-person training at the initiation of the study or the site by the PI of the study (AKP). In 2014 (NHS #3, NCT02738281), the data capture forms underwent a major revision which included asking caregivers to rank the top 3 concerns for the affected participant at each visit (described below). From 2014 to March 2021, a total of 994 unique participants were enrolled and assessed, with the majority being participants with the diagnosis of classic, or typical RTT (Table 1). For the work presented here, we excluded from analyses participants with duplications of *FOXG1* (*n* = 3), due to the small number of participants, and those grouped into the diagnostic category “other”, which was genetically and clinically heterogeneous (e.g., people with *MECP2* mutations who do not meet clinical criteria for RTT; people with mutations in genes other than *MECP2*, *CDKL5*,* FOXG1*; variants of unknown significance). Ultimately, we analyzed data on 925 participants, with *n* = 641 having classic RTT, *n* = 84 having atypical RTT, *n* = 74 with *MECP2* duplication syndrome (MDS), *n* = 67 with CDKL5 deficiency disorder (CDD), and *n* = 59 having FOXG1 syndrome (FS). The full breakdown of the participants, sex, and age groups is provided in Table 1. The *MECP2* mutation (or mutation groups) distributions for classic and atypical RTT are provided in Additional file 1: Table S1.

**Table 1:** Number of participants by diagnostic category and age bins

Diagnosis	Total	Female	Male	Age (years)									
				< 1	1 to 3	3 to 5	5 to 10	10 to 15	15 to 20	20 to 25	25 to 30	30 to 40	> 40
Classic RTT	641	638	3	0	45	73	145	119	99	58	40	46	16
Atypical RTT	84	80	4	0	8	16	16	9	16	7	2	8	2
MDS	74	7	67	1	15	14	17	13	6	2	5	1	0
CDD	67	55	12	6	14	13	19	8	5	1	1	0	0
FS	59	35	24	6	16	8	20	6	2	0	0	1	0
Total	925	815	110	13	98	124	217	155	128	68	48	56	18

The racial and ethnic demographic information for the participants, as well as the parental educational level, employment, and household income, is provided in Table 2. Most participants were White, non-Hispanic (77.3%), with the next largest racial/ethnic group identified as White, Hispanic (9.9%). The majority of mothers and fathers had some college or more education (81.7% and 70.1% respectively) and were employed outside the home (55.9% and 79.5% respectively). A significant percentage of the participant’s household income was over $100,000 per year (38.6%), and only a small percentage of household income was less than $20,000 per year (4%). Within Table 2, “not applicable” was selected for parent education and household income for participants who had no living parents and lived in residential care facilities. Additionally, “not applicable” was selected for parent education if only one parent existed to provide an educational level.

**Table 2:** Demographic information

Participants (*n* = 925)		Number (%)	
		Ethnicity	
		Non-Hispanic	Hispanic
**Race**	White	715 (77.3)	92 (9.9)
	African American/Black	31 (3.4)	2 (0.2)
	Asian	28 (3)	3 (0.3)
	Native American	5 (0.5)	1 (0.1)
	Hawaiian/Pacific Islander	1 (0.1)	0 (0)
	More than one race	26 (2.8)	5 (0.5)
	Not reported	4 (0.4)	8 (0.9)
	Refused	2 (0.2)	2 (0.2)
**Parents**		**Mother**	**Father**
**Education**	Advanced degree	188 (20.3)	191 (20.6)
	Bachelor’s degree	293 (31.7)	235 (25.4)
	Some college, no bachelor’s degree	275 (29.7)	223 (24.1)
	High school diploma or GED	118 (12.8)	152 (16.4)
	No high school diploma or GED	23 (2.5)	28 (3)
	Not applicable	25 (2.7)	86 (9.3)
	Declined	3 (0.3)	3 (0.3)
	Unknown	0 (0)	7 (0.8)
**Employment**	Employed	517 (55.9)	735 (79.5)
	Homemaker	313 (33.8)	12 (1.3)
	Retired	26 (2.8)	44 (4.8)
	Student	11 (1.2)	4 (0.4)
	Disabled	7 (0.8)	11 (1.2)
	Unemployed	25 (2.7)	19 (2.1)
	Unknown	26 (2.8)	100 (10.8)
**Household income**	Less than $20,000	37 (4)	
	$20,000–$39,999	82 (8.9)	
	$40,000–$59,999	113 (12.2)	
	$60,000–$79,999	119 (12.9)	
	$80,000–$99,999	93 (10.1)	
	$100,000 or more	357 (38.6)	
	Declined	109 (11.8)	
	Not applicable	15 (1.6)	

### Creation of top caregiver concern list

To capture top caregiver concerns, at each visit the caregiver was asked to identify the top 3 concerns (First Concern, Second Concern, Third Concern) from a list of 21 concerns (described below) as well as having the option of selecting “Other” and entering a free text response. The rank order was not predetermined by the investigators but selected by the caregivers. The list of 21 concerns was developed via a review of the published literature for RTT and discussion amongst expert clinicians to create a list of potential concerns that represented ‘Disease Defining Concepts’ such as impaired hand use, communication difficulties, problems walking, and repetitive hand movements, and other commonly observed clinical features such gastrointestinal/nutritional issues (difficulty chewing and swallowing, poor weight gain, gastroesophageal reflux, constipation), breathing dysrhythmias, sleep problems, seizures, and behavioral issues (anxiety, aggression, self-abusive behaviors), as well as others (e.g., teeth grinding). This list was discussed with caregivers of people with RTT associated with the International Rett Syndrome Foundation to provide input if any potential concerns were not included and a final list of choices was incorporated into the revised data collection forms. The choices are shown in Table 3. The caregivers also had the option to select “other” and enter a free text description of the concern. Caregivers selected “other” for First Concern 32 times (3.5%), for Second Concern 59 times (6.4%), and for Third Concern 72 times (7.8%). With a total number of “other” selected 163 times (5.9% of all entries). The free text answers for the “other” choices were reviewed manually (by JLN), to identify free text responses that fell into the pre-specified choices (e.g., free text “hyperventilation” which fits into rapid breathing or breath holding while awake). The remaining free text responses were grouped into 15 additional clinical categories (e.g., “abdominal pain”, “gall bladder” and “vomiting” each contributed to the additional “Other GI” category). A table of the free text entries and reclassification categories is provided in Additional file 2: Table S2. The reclassification into appropriate prespecified categories or created categories was reviewed and agreed upon by the other authors. This resulted in a final total of 36 distinct concern categories (Table 3).

**Table 3:** Top concern categories

**Prespecified concern choices**
Abnormal movements (other than hand stereotypies)
Abnormal walking/balance issues
Aggressiveness towards others
Air swallowing/bloating/excessive gas
Anxiety
Constipation
Frequent infections
Gastro-esophageal reflux
Lack of effective chewing or swallowing
Lack of effective communication
Lack of hand use
Poor weight gain
Problems with Sleep
Rapid breathing or breath holding while awake
Repetitive hand movements
Scoliosis
Screaming episodes
Seizures
Self-abusive behavior
Teeth grinding (while awake)
Vision
Other (please specify)
**Created terms**
Attention/cognition/developmental delay/ID
Drooling/spitting
Dystonia/rigidity/contractures
Fatigue/lethargy/energy
GU issues
Hypotonia
None indicated
Other autonomic
Other behavior
Other GI
Other health issue
Other musculoskeletal
Pain issues
Respiratory/pulmonary
Therapy issues

### Creation of weighted top concerns

To generate a list of top concerns, we analyzed these responses from the baseline visit for participants (no longitudinal evaluation from subsequent visits was performed). To account for the relative importance of the concerns, we weighted each concern based on the rank order reported by the caregiver (weighted rank = 1/rank order). Thus, for each participant, the First Concern received a weighted rank of 1, the Second Concern a weighted rank of 0.5 and Third Concern a weighted rank of 0.33 for each patient. The weighted scores for each category were summed for each diagnostic category, and for classic RTT across age groups, severity groups, and mutation groups. A rank order for the top concern categories for each group was then created (top rank = higher weighted score), and the percentage for each concern category was calculated by dividing the weighted category score by the total of all weighted category scores for a given grouping that was analyzed. The group analysis was conducted on all participants for Classic RTT, as well as by age bins, severity, and *MECP2* mutations. Group analysis was also performed based on diagnostic categories (classic RTT, atypical RTT, MDS, CDD, and FS). Analysis by age bins, severity, and specific genetic mutation was only conducted for classic RTT, as the overall number of individuals in the other disorders was limited when broken into further subgroups.

### Evaluation of weighted top concerns in classic RTT

For classic RTT, the weighted top concerns rankings were compared by calculating the 95% confidence interval (CI) for each concern using the standard deviation calculated from a binomial distribution, identifying those concerns whose CI included zero. Significant differences for pairwise comparisons between weighted top caregiver concerns for classic RTT are reported at the *p* < 0.05 level.

Weighted top concerns for classic RTT were analyzed by age, clinical severity, and common RTT-causing *MECP2* mutations [6, 7]. Clinical severity was assessed using two clinician-assessed measures (performed by physician investigators), the Clinical Global Impression-Severity (CGI-S) and the RTT Clinical Severity Score (CSS) [34]. The CGI-S is a clinician assessment of overall clinical severity scored on a seven-point Likert score (1 = normal function, 7 = worst level of function) based on established RTT-specific anchors [34]. The CSS is a clinical rating scale composed of 13 elements, each having Likert scores from 0–4 or 0–5, with a range of total CSS score from 0 to 58 (0 = normal, 58 = most severe involvement) [6]. Rater training o was conducted by in-person site visits by AKP (PI of NHS).

### Comparison of top caregiver concern to clinician assessment of clinical features in classic RTT

To evaluate the relationship of the First Caregiver Concern (not weighted) at baseline for an individual to clinical features noted by a physician, we compared the listed first concern to individual CSS item scores related to language, seizures, and hand use at the baseline visit. The percentage of caregivers who listed Lack of Effective Communication (Communication), Seizures, and Lack of hand use (Hand Use) for each item score for CSS Language, CSS Seizure, and CSS Hand Use score was calculated.

### Comparison of weighted top caregiver concerns across disorders

The weighted top concerns at the baseline visit for classic RTT were compared to those for Atypical RTT, MDS, CDD, and FS. The smaller number of participants in these other diagnostic categories (Table 1) precluded further analysis by age. For atypical RTT, we also developed weighted concerns for those regarded as being “milder” or more “severe” than classic RTT, as people grouped into the atypical RTT category have a bimodal severity distribution when assessed with the CSS [34]. Based on this, a cutoff of CSS < 18 was used to define the “mild” atypical group and CSS > 18 was used to define the “severe” atypical group.

### Evaluation of top caregiver concern relative to caregiver impression of change

During study visits, caregivers were asked their impression of whether their child had improved, remained unchanged, or worsened over the last six months using a 5-point Likert scale (much improved, improved, unchanged, worse, or much worse), and indicate the reason for their impression. In contrast to the top weighted caregiver concern comparisons outlined above which utilized only baseline visit data, for this analysis, we did not restrict to only the baseline visit data but used the entire longitudinal data set including repeated visits. We evaluated the reasons provided by the caregivers for their impression of any improvement (much improved or improved) or any worsening (much worse or worse) to identify the top caregiver reason for the impression of change for participants with classic RTT, MDS, CDD, and FS. We then calculated the frequency (number, percentage) of times the first listed caregiver concern was listed as effective communication or seizures based on the caregiver's impression of improvement or worsening for each diagnostic group.

## Results

### Top caregiver concerns in classic RTT

**Fig. 1:**
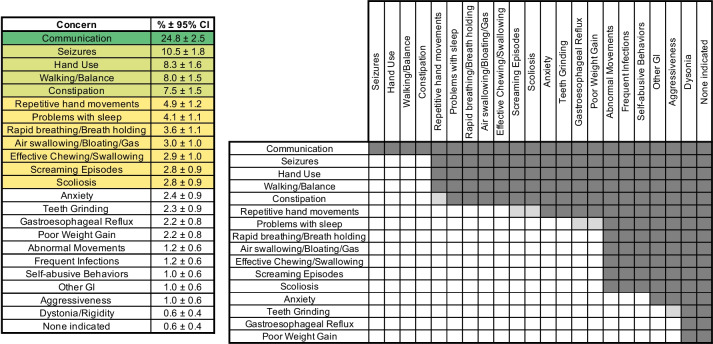
Weighted top caregiver concerns for classic RTT. The percentage of each weighted top caregiver concern is presented on the left with 95% CI, with groupings shaded as described in the text. The right side of the figure shows significant differences between weighted concerns as shaded cells (dark gray, *p* < 0.05; light gray *p* < 0.10)

The top 5 weighted concerns reported by caregivers for people with classic RTT (Fig. 1) are (1) lack of effective communication; (2) seizures; (3) lack of hand use; (4) abnormal walking/balance; and (5) constipation. Notably, these top 5 weighted concerns were identified as one of the top 3 listed concerns (meaning the caregiver selected the concern as either the 1st, 2nd, or 3rd concern) by > 25% of caregivers (communication 60.2%; seizures 27.9%; hand use 27.8%; walking balance 25.6%; constipation 25.3%), whereas the subsequent top weighted concerns were identified by < 17% of caregivers as one of the top 3 listed concerns. Figure 1 displays the weighted concerns whose 95% CI are above zero on the left, with the pairwise differences between concerns shown on the right. The concerns can be placed into “groups” of concerns that are not statistically different from each other but different from other concern groups. Lack of effective communication (communication) stands out as significantly different than all other concerns and is considered group 1 (dark green in Fig. 1). Group 2 (seizures, hand use, walking/balance, and constipation, light green in Fig. 1), represents important caregiver concerns with percentages ranging from 7.5 to 10.5%, and different from subsequent groups. Group 3 contains concerns meaningful in classic RTT (repetitive hand movements, sleep problems, breathing abnormalities, etc.) with percentages ranging from ~ 3 to 5% (yellow in Fig. 1). Group 4 consists of concerns with percentages between 2 and 2.5%, and group 5 represents concerns that overall are relatively low frequency in classic RTT (~ 1%, white in Fig. 1). Overall, the top 3 groups of caregiver concerns likely represent the most relevant concerns in classic RTT (overall frequency between ~ 3% and 25%) and align well with known clinical problems observed in RTT [3].

### Variation in top caregiver concerns in classic RTT between age groups

**Fig. 2:**
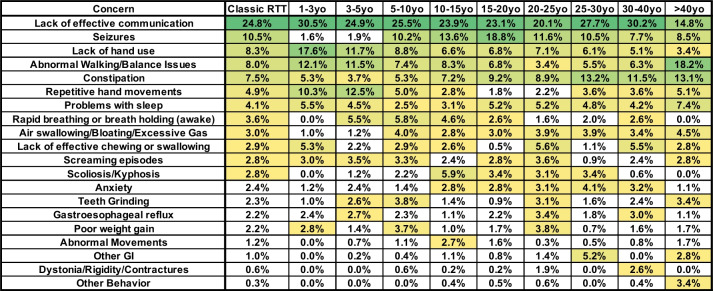
Weighted top caregiver concerns for classic RTT vary by age. Top weighted concerns are listed on the left, with the order presented representing the rank order for all people with Classic RTT. Age bins are shown in subsequent columns. The heatmap color shows the highest ranked concerns as dark green (as in group 1 in Fig. 1), with intermediate ranked concerns as light green (as in group 2 in Fig. 2), and lower frequency concerns as yellow (as in group 3 in Fig. 1, with a lower cutoff of 2.5%). Concerns with weighted rank percentages below 2.5% are in white. Concerns are included only if at least one cell for concern had a percentage above 2.5% within any of the age bins. Abbreviations: GI = gastrointestinal

While assessment of the weighted caregiver concerns across all participants with classic RTT identified the most relevant issues in classic RTT, there was variation in top caregiver concerns in different age groups, especially those concerns identified with frequency above 2.5% (groups 1–3) in the overall classic RTT cohort (Fig. 2). Effective communication remained a high-level concern across all age groups; however, within the oldest age group, concern about ambulation became more frequent. Seizures, the second-most overall concern across the classic RTT group (with a percentage of 10.5%), was a minor concern under age 5 (1.6–1.9%), climbed in frequency after 5 years old, peaked in the 15–20-year-old group, and declined in the over 20 years old age bins while remaining a high-level concern (> 8%). This pattern of caregiver concern is consistent with clinical observation of the peak period of seizure onset and severity [35]. Lack of hand use was a frequent concern across all age groups but declined in frequency with age despite the lack of notable improvement in hand function in older age groups [36]. Caregiver concern about constipation, a common problem [37], generally increased in importance with age, especially in the older age groups. Repetitive hand movements [36] were a frequent concern throughout most of the age groups, with a peak during the first 5 years, but declined to lower frequency (< 2.5%) between 15 and 25 years old with a subsequent progressive increase in older age groups. Rapid breathing or breathholding was non-existent as a concern until age 3, increased through age 15 and then declined to non-existent in the oldest group, following expected trends observed for the incidence of breathing abnormalities [38]. Similarly, air swallowing/bloating only became a major concern after 5 years old. Concern about scoliosis remained low in younger ages, peaked in the 10–15-year-old group, and then declined, consistent with the timing of marked progression of scoliosis in classic RTT [5]. Notably, concerns that in the overall group fell into the ~ 2% range (group 4 in Fig. 1), such as anxiety, teeth grinding, and gastroesophageal reflux, increased in frequency of concern in various age groups; however, they remained below 5% throughout the age groups. Thus, while the overall caregiver concerns for classic RTT from the entire cohort are useful, especially the high-frequency caregiver concern groups (groups 1–3), consideration for the age-related differences in the relative frequency of caregiver concerns is important.

### Caregiver concerns in classic RTT based on MECP2 mutation

**Fig. 3:**
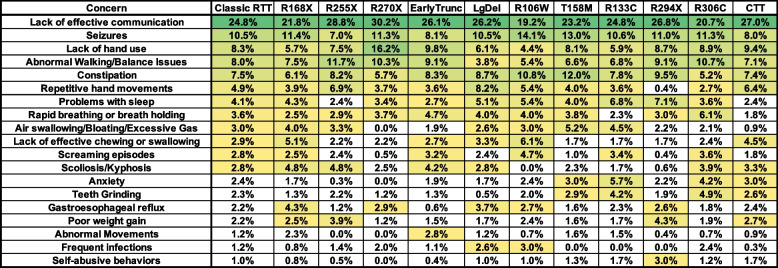
Weighted top caregiver concerns for classic RTT across *MECP2* genotypes. Top weighted concerns are listed on the left, with the order presented representing the rank order for all people with classic RTT. *MECP2* mutation groups are shown in subsequent columns, arranged with more severe mutations on the left. Abbreviations: EarlyTrunc = Early Truncations; LgDel = Large Deletions; CTT = C-terminal truncations. Heatmap color, concern presentation (> 2.5% in at least one cell), and other abbreviations are as in Fig. 2

Based on known genotype–phenotype relationships [6, 7], we compared variation in caregiver concerns for Classic RTT across the common, recurrent *MECP2* mutations (R168X, R255X, R270X, R106W, T158M, R133C, R294X, R306C) as well as mutation groupings that cause similar molecular disruption of the *MECP2* gene (early truncations, large deletions, C-terminal truncations [CTT]) compared to the combined caregiver concerns for people with Classic RTT (Fig. 3). The top 5 concerns (groups 1–2 in Fig. 1) remained frequent concerns (> 3%) across the mutation groups. Within the overall group 3 concerns (2.5–5%), some notable changes were observed, with repetitive hand movements dropping significantly in R924X, air swallowing dropping in R270X and CTT, screaming episodes dropping in R270X and R294X, and scoliosis dropping in R106W and R294X. In contrast, some of the caregiver concerns identified in the lower range within the overall Classic RTT cohort (between 1 and 2.5%), were increased in frequency within specific mutation groups. For example, behavioral problems such as anxiety and self-abusive behaviors were more frequently raised as caregiver concerns in milder mutations such as R133C, R294X, and R306C, concordant with the clinically observed increased rates of behavioral problems in less severely affected individuals with classic RTT [39], whereas frequent infections were more common concerns in severe mutations such as large deletions and R106W.

### Caregiver concerns in classic RTT vary by clinical severity

**Fig. 4:**
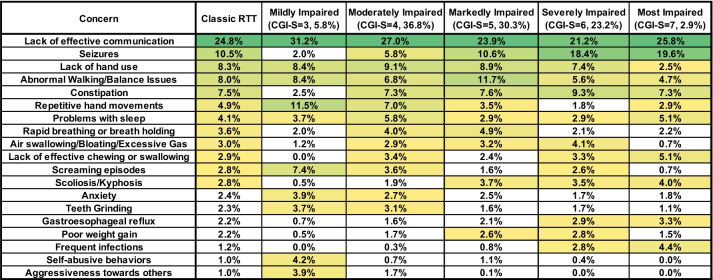
Weighted top caregiver concerns for classic RTT across CGI-S scores. Top weighted concerns are listed on the left, with the order presented representing the rank order for all people with Classic RTT. CGI-S are shown in subsequent columns. Percentages of people in each CGI-S group are shown in the header. Heatmap color, concern presentation (> 2.5% in at least one cell), and abbreviations are as in Fig. 2

To assess whether caregiver concerns varied by clinical severity, we evaluated top caregiver concerns in different severity groups as determined by clinician-assessed severity using the Clinical Global Impression–Severity (CGI-S) and RTT Clinical Severity Score (CSS). Within the severity categories defined by the CGI-S, lack of effective communication remained the top concerns across all severity groups (Fig. 4). Within the group 2 concerns (seizures, hand use, walking/balance, and constipation), it is notable that seizures and constipation were low-frequency concerns in the mildest severity group (CGI-S = 3), but became progressively higher frequency concerns with increasing severity. Lack of hand use was a constant high-frequency concern throughout most of the severity range (CGI-S = 3–6), but unexpectedly dropped to only 2.5% in the most impaired group (CGI-S = 7), despite the fact that people within this severity group have the most overall impaired hand function. Abnormal walking/balance issues were a high-frequency concern across the severity range, with a peak in the markedly impaired group (CGI-S = 5) and decline in the more severely affected groups (CGI-S = 6–7). Concern about repetitive hand movements was greatest in the mildest severity groups (CGI-S = 3–4) and declined in the more severely affected groups (CGI-S = 5–7). In contrast, rapid breathing or breath holding and Air swallowing/bloating were more frequent in the middle severity groups (CGI-S = 4–6), which represent the bulk of people with classic RTT, and lower in the mildest (CGI-S = 3) and most severe (CGI-S = 7) groups. Behavioral features (screaming episodes, anxiety, self-abusive behaviors, aggressiveness) and bruxism concerns were increased in the mildest group (CGI-S = 3), but were very low in the most severe group (CGI-S = 7), consistent with the observation that behavioral issues are more prominent in less severely affected individuals [39]. On the other hand, concerns such as scoliosis, gastroesophageal reflux, poor weight gain, and frequent infections were low-frequency concerns in the milder severity groups (CGI-S = 3–4), but were meaningful concerns (percentage > 2.5%) in more severely affected groups (CGI-S = 6–7).

**Fig. 5:**
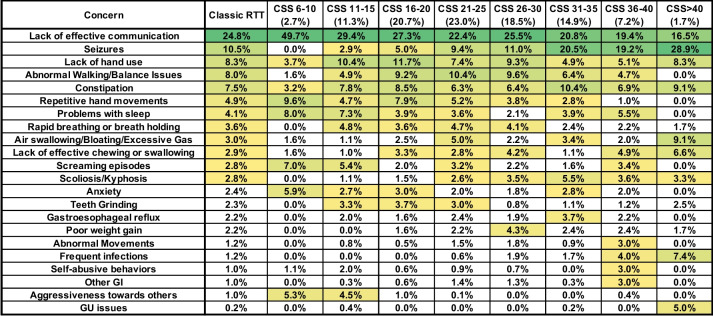
Weighted top caregiver concerns for classic RTT across CSS scores. Top weighted concerns are listed on the left, with the order presented representing the rank order for all people with classic RTT. CSS are shown in subsequent columns, arranged in groups from least to most severe. Percentages of people in each CSS group are shown in the header. Heatmap color, concern presentation (> 2.5% in at least one cell), and abbreviations are as in Fig. 2, with additional abbreviation: GU = Genitourinary

The comparison of caregiver concerns with severity assessed using the CSS broadly showed similar results as with severity assessed with the CGI-S, but there are some notable differences (Fig. 5). Effective communication remained a high-frequency concern across all CSS groups; however, the percentage in the mildest group (CSS 6–10) was double that for the overall classic RTT cohort (49.7% vs 24.8%) and in the most severe group (CSS > 40), the percentage of Communication concern dropped to 16.5%, below that for seizures in this group. Concern about seizures showed the same pattern in CSS severity groups as in CGI-S groups, with seizures not being a concern in the mildest group (CSS 6–10), but progressively became more frequent with increasing CSS severity. Similarly, concern about walking/balance was highest in the middle CSS severity groups (CSS 16–30), low in the mildest (CSS 6–10), and non-existent in the most severe (CSS > 40), consistent with the pattern observed in the CGI-S severity groups. Repetitive hand movement concerns also showed the same pattern in the CSS severity groups as observed in CGI-S severity groups, being most prominent in the less severe CSS groups and dropping in the most severe CSS groups (CSS 36–40 and CSS > 40). In contrast, the decline in concern for Hand Use in the most severe CGI-S group was not observed in the CSS severity groups, with the frequency of concern related to hand use remaining high in the most severe CSS group (CSS > 40). While behavioral concerns such as screaming episodes, anxiety, and aggressiveness were high in mild CSS severity groups and low in the most severe CSS severity groups, unexpectedly concern for self-abusive behaviors was low in all CSS severity groups except the second most severe group (CSS 36–40). As seen in the analysis based on CGI-S severity, medical concerns such as scoliosis, frequent infections, and genitourinary (GU) issues increased with CSS severity.

### Comparison of caregiver concerns to assessments of clinical features

To evaluate the relationship of the First Caregiver Concern (not weighted) for an individual to clinical features, we compared the First (number 1) listed caregiver concern to individual CSS item scores related to language, seizures, and hand use. Overall, 35.3% (*n* = 226) caregivers reported effective communication (communication) as the First Concern, 12.6% (*n* = 81) listed Seizures as the First Caregiver Concern, and 5.5% (*n* = 35) listed lack of hand use (hand use) as the First Caregiver Concern (Table 4). Within each of these three First Caregiver Concerns, we calculated the number (and percentage) that were given CSS scores on the CSS items Language, Seizure, and Hand Use (Table 4).

**Table 4:** Comparison of individual CSS item scores to number one caregiver concern

		Number 1 concern		
		Communication (*n* = 226, 35.3%)	Seizures (*n* = 81, 12.6%)	Hand Use (*n* = 35, 5.5%)
		CSS score (*n*, %)	CSS score (*n*, %)	CSS score (*n*, %)
**CSS Language**	0—Preserved, contextual	0 (0.0%)	1 (1.2%)	0 (0.0%)
	1—Short phrases only	2 (0.9%)	1 (1.2%)	0 (0.0%)
	2—Single words	26 (11.5%)	5 (6.2%)	0 (0.0%)
	3—Vocalization, babbling	140 (61.9%)	41 (50.6%)	27 (77.1%)
	4—Screaming, no utterances	58 (25.7%)	33 (40.7%)	8 (22.9%)
**CSS Seizures**	0—Absent	126 (55.8%)	1 (1.2%)	23 (65.7%)
	1— < Monthly	42 (18.6%)	10 (12.3%)	4 (11.4%)
	2— < Weekly to monthly	21 (9.3%)	10 (12.3%)	4 (11.4%)
	3—Weekly	15 (6.6%)	22 (27.2%)	2 (5.7%)
	4—More than weekly	10 (4.4%)	10 (12.3%)	1 (2.9%)
	5—Daily (intractable)	12 (5.3%)	28 (34.6%)	1 (2.9%)
**CSS Hand Use**	0—Conserved	29 (12.8%)	3 (3.7%)	4 (11.4%)
	1—Acquired on time, partially conserved	37 (16.4%)	7 (8.6%)	7 (20.0%)
	2—Acquired late, partially conserved	25 (11.1%)	7 (8.6%)	1 (2.9%)
	3—Acquired and lost	120 (53.1%)	56 (69.1%)	20 (57.1%)
	4—Never acquired	15 (6.6%)	8 (9.9%)	3 (8.6%)

Legend: The First Caregiver Concern for Classic RTT is shown along the top, with the number (and percentage) of caregivers listing the concern presented (out of a total of *n* = 641 participants). The CSS items assessing Language, Seizures, and Hand Use is presented on the left side of the table, with increasing CSS item scores representing increased severity (as shown in the description of the score levels). The number (and percentage) of CSS item scores for each First Concern is presented within the cells. The total numbers and percentages sum within each First Concern within the column for each CSS item

When communication was indicated as the First Caregiver Concern, a large percentage of participants had a CSS Language score = 3 (vocalization, babbling), but the percentage dropped at the most severe CSS Language score = 4 (screaming, no utterances). However, this pattern was also observed in the CSS Language score distribution when hand use was indicated as the First Caregiver Concern. In contrast, when the First Caregiver Concern was seizures, a similar percentage of participants had CSS Language scores of 3 or 4. Interestingly, the percentage of participants with more preserved language function (CSS Language scores of 0, 1, or 2) in the group with the First Caregiver Concern was 12.4%, compared to 0% in the group that indicated hand use as the First Caregiver Concern, suggesting that communication is a larger concern for caregivers when their child has more language skills.

A similar pattern is observed when the First Caregiver Concern was Hand use, with a large percentage of participants having a CSS Hand Use score = 3 (acquired and lost), but a significant decline in the percentage of participants having a CSS Hand Use score = 4 (never acquired). Again, this pattern of change in the percentage of individuals in the two most severe CSS Hand Use was not unique to the group that had the First Caregiver Concern of Hand Use but was also present in the groups that had the First Caregiver Concern of Communication or Seizures. The percentage of participants with some level of hand function (CSS Hand Use scores of 0, 1, or 2) were similar when the First Caregiver Concern was hand use or communication (34.3% and 40.3% respectively), but interestingly was lower when the First Caregiver Concern was Seizures (20.9%).

The most dramatic difference between the First Caregiver Concern groups was observed in the percentage of participants who did not have seizures (CSS Seizure score = 0, absent). When the First Caregiver Concern was Communication or Hand Use, a large percentage of participants had a CSS Seizure score of 0 (55.8% and 65.7% respectively). In contrast, when the First Caregiver Concern was Seizures, only 1.2% had a CSS Seizure score of 0. Similarly, 46.9% of participants in the Seizures First Caregiver Concern group had severe CSS Seizure scores (CSS Seizure score of 4 or 5), whereas the percentage of participants with severe CSS Seizure scores was much lower in the communication (9.7%) or the hand use (5.8%) First Caregiver Concern group. Thus, the presence of seizures drives caregivers to list seizures as the First Concern, despite the overall poor skills in this group in language or hand use.

### Caregiver concerns in atypical RTT

**Fig. 6:**
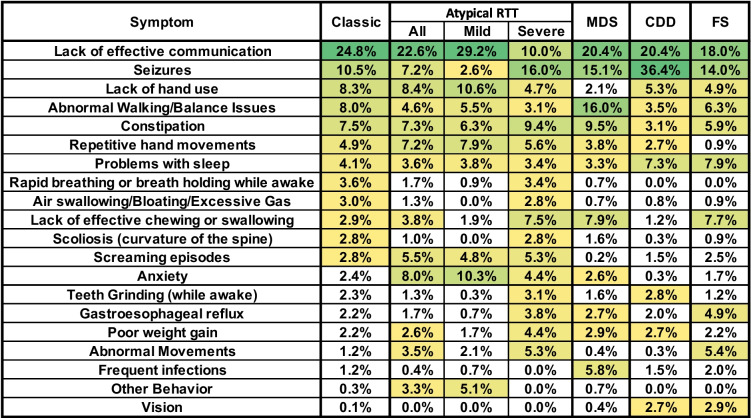
Weighted top caregiver concerns for atypical RTT, MDS, CDD, and FS. Top weighted concerns are listed on the left, with the order presented representing the rank order for all people with classic RTT. For atypical RTT, total results for all people with atypical RTT are shown, as well as those considered “mild” (CSS < 18) and those considered “severe” (CSS > 18). Heatmap color, concern presentation (> 2.5% in at least one cell), and abbreviations are as in Fig. 2

The top concerns for caregivers of individuals with Atypical RTT were generally similar to those reported in classic RTT, especially in the highest frequency concerns (Fig. 6), but some lower frequency concerns for classic RTT, such as rapid breathing or breath holding, Air swallowing/bloating, scoliosis, and anxiety were different between classic RTT and the entire atypical RTT group. However, atypical RTT is composed of individuals who are milder and more severely affected than classic RTT, as shown by the bimodal distribution of total CSS scores in atypical RTT, mild atypical RTT having a total CSS score < 18 and Severe Atypical RTT having a total CSS score > 18 [34]. The pattern of caregiver concerns is markedly different between these groups of Atypical RTT, with Mild Atypical RTT having a decreased frequency of Caregiver Concerns for a number of items such as seizures, rapid breathing/breath holding, and scoliosis, but increased frequency for behavioral issues such as anxiety and other behavioral issues. For people with severe atypical RTT, caregivers indicated increased concerns in areas such as seizures, abnormal movements, and GI issues (lack of effective chewing/swallowing, gastroesophageal reflux, poor weight gain).

### Comparison of caregiver concerns between Classic RTT and RTT-related disorders

Top concerns were compared across RTT-related disorders including MDS, CDD, and FS (Fig. 6). Lack of effective communication remained the top-weighted concern for both MDS and FS, but for CDD seizures become the top-weighted concern, with more than 20% higher than for Classic RTT. This reiterates the known increase in overall seizure burden in people with CDD [22, 40]. Lack of hand use remained a frequent concern in CDD and FS but dropped markedly for MDS. In contrast, Walking/balance concerns increased in MDS. Caregivers did not endorse Repetitive hand movements as a frequent concern in FS. Some concerns in group 3 for classic RTT such as rapid breathing/breath holding, air swallowing/bloating, scoliosis, and screaming episodes were found at low frequency in MDS, CDD, and FS, whereas lack of effective/chewing had a higher frequency in MDS and FS than classic RTT. Other low-frequency concerns in classic RTT (~ 1%) were more frequently endorsed in other disorders, consistent with known issues in these disorders. For example, in MDS there was a higher frequency of caregiver concern for frequent infections, a noted problem in MDS [41–43], and in FS there is an increase in caregiver concern for abnormal movements [44]. Concerns about vision were present in people with CDD and FS, both of which have reported issues with cortical visual impairment [22, 45, 46].

### Caregiver impression of change: reasons and top concern

At each visit, caregivers provided a global impression of whether they felt that their child had improved, worsened, or remained unchanged, to identify the main reason for their overall global impression for improvement or worsening. For all visits, a significant number of caregivers felt that there was no change for their child, although this varied across disorders (Table 5). The most frequent caregiver reason provided for improvement for all disorders (classic RTT, MDS, CDD, FS) was communication (Table 5), although variation was noted between the disorders. When caregivers reported improvement, the first listed top concern for classic RTT, MDS, and FS was lack of effective communication; however, for CDD Seizures was most frequent first concern (47%) with lack of effective communication still being a frequent first concern (33%). The top caregiver-reported reason for worsening in all disorders was seizures, ranging from 19% for classic RTT to 63% for CDD (Table 5). When caregivers reported worsening, the first listed caregiver concern for all disorders was seizures. The concern of lack of effective communication remained a high-frequency caregiver concern in classic RTT but dropped dramatically in the other disorders. Overall, the top caregiver reason for improvement was communication and for worsening was seizures. The number one (first listed) caregiver concern aligned with the caregiver impression of change, with the notable exceptions that when improvement was noted, caregivers of participants with CDD listed seizures as their first concern although identified communication as the reason for improvement, and when caregivers of participants with classic RTT felt their child was worse the frequency of first concern was very similar for seizures and lack of communication although the top reason for worsening was seizures.

**Table 5:** Caregiver impression of change

Caregiver impression of change	RTT (*n* = 1282)	MDS (*n* = 163)	CDD (*n* = 147)	FS (*n* = 141)
Unchanged	51.6% (662/1282)	32.5% (53/163)	38.1% (56/147)	43.3% (61/141)
Improved	27.2% (349/1282)	47.2% (77/163)	48.0% (72/147)	44.0% (62/141)
Reason for improvement: communication	36.7% (128/349)	29.9% (23/77)	29.2% (21/72)	17.7% (11/62)
*First caregiver concern: communication*	*29.5%* *(103/349)*	*41.6%* *(32/77)*	*33.3%* *(24/72)*	*27.4%* *(17/62)*
*First caregiver concern: seizures*	*7.7%* *(27/349)*	*10.4%* *(8/77)*	*47.2%* *(34/72)*	*17.7%* *(11/62)*
Worse	21.1% (271/1282)	20.2% (33/163)	12.9% (19/147)	12.8% (18/141)
Reason for worsening: seizures	19.2% (52/271)	42.4% (14/33)	63.2% (12/19)	22.2% (4/18)
*First caregiver concern: seizures*	*21.4%* *(58/271)*	*54.5%* *(18/33)*	*78.9%* *(15/19)*	*38.9%* *(7/18)*
*First caregiver concern: communication*	*18.1%* *(49/271)*	*3.0%* *(1/33)*	*5.3%* *(1/19)*	*5.6%* *(1/18)*

Legend: The number of visits evaluated (in parentheses) for the different disorders is listed across the top. The percentage of visits the caregiver noted an impression of change as unchanged, improved, or worse is noted for each disorder (number of visits with caregiver impression/total number of visits). For visits the caregiver impression was improved or worse, the top reason for the impression is stated, with the percentage (number of visits with the top reason/total number visits with stated impression). Similarly, the percentage of visits in which the caregiver indicated the top concern was seizures or communication is shown for visits the caregiver impression was improved or worse is presented (number of visits with first concern/total number of visits with stated impression)

## Discussion

Analysis of the top caregiver concerns in people with RTT and Rett-related disorders from a large natural history study provided important information relevant to the design and selection of clinical outcome measures. We found that the top concerns of caregivers of individuals with Classic RTT generally align with common clinical features, particularly related to functional skills lost in RTT (communication, walking, hand use). The top-weighted caregiver concern was the child’s inability to effectively communicate. This is not surprising as communication is fundamental to interpersonal connections and the loss of both hand skills and spoken language profoundly impairs RTT individuals’ ability to effectively communicate. Seizures and constipation are in the top 5 weighted caregiver concerns for classic RTT emphasizing the prevalence and importance of these clinical problems in RTT [3]. The top 5 weighted caregiver concerns represent key domains to be assessed in a clinical outcome measure, as they also were all identified as one of the top 3 concerns by more than 25% of caregivers. However, consideration of other caregiver concerns within group 3 (Fig. 1) should not be discounted, as they are relevant clinical issues in classic RTT and are more concerning within specific age and clinical severity groups.

Top caregiver concerns in classic RTT vary based on age, *MECP2* mutation, and clinical severity, with changes following expected patterns related to the relative prevalence of specific clinical issues that change with age or clinical severity [35, 36, 38, 39]. For example, seizures do not become concerning until the age range at which seizures are prevalent in people with Classic RTT [35]. Interestingly, although functional skills such as hand use and ambulation are more impaired in severely affected individuals and do not improve in older age individuals, the frequency of caregiver concern for these functional skills as higher concerns dropped in the most severely affected individuals and in older age bins. This unexpected result may indicate that caregivers of older or more severely affected individuals have adjusted expectations with regard to functional impairment and have developed larger concerns related to pressing medical issues. Additional work is needed to assess caregiver expectation within these groups to determine if this hypothesis is correct.

While broad similarities were identified in the top concerns of caregivers of people with Atypical RTT and classic RTT, differences were noted when the atypical RTT group was split into “milder” and “severe” groups. We observed a rise in the frequency of concerns related to behavior in the milder group and seizures in the severe group. This pattern is consistent with that observed in classic RTT when analyzed based on clinical severity, reflecting observed variation in the prevalence and severity of specific clinical problems. For example, individuals with milder motor impairment (such as less-severely affected classic RTT or mild atypical RTT) display more behavioral problems [47, 48].

As hypothesized, comparison of top caregiver concerns between RTT and other RTT-related disorders identified consistent concerns between these disorders in some concerns but also revealed differences aligned with known differences in clinical features in these disorders. Communication was a top concern across all disorders, reflecting the marked impairment in communication ability in all these disorders. Notably, communication was the top concern in every disorder except for CDD, in which seizures become the overall top caregiver concern. This is concordant with the relative seizure burden and impact in CDD relative to the other disorders [17]. Similarly, frequent infections was a meaningful concern only in caregivers of people with MDS, reflecting the higher rate of infections in this population relative to the other disorders [41–43]. Thus, while these disorders display overlap in some clinical phenotypes, differences in caregiver concerns between these disorders demonstrate that consideration of the specific clinical phenotypes within the different disorders is critically important.

Identifying patients’/caregivers' major concerns that have the greatest impact on daily life is a priority and mandate from the FDA [29, 30] in the development of meaningful outcome measures for clinical trials. Furthermore, the FDA acknowledges that utilization of caregiver information may be needed for affected individuals with cognitive limitations [30]. Failure of a therapy to modify the top concerns of patients/caregivers of individuals may result in regulatory advisory panels (FDA/EMA) to not endorse an investigational product for approval. This patient-focused approach requires outcome measures that can capture the breadth of a disease’s impact across a heterogeneous range of severity, age, and mutation. In rare disorders with a small number of participants, robust outcome measures that capture the heterogeneity of diseases are needed to achieve measurable outcomes in clinical trials. For example, our data demonstrates that a product that does not impact seizures could still be considered to have a meaningful impact if communication improves. Thus, broad measures assessing multiple clinical issues are important to capture in rare disease outcome measures.

While this work evaluated concerns captured from a large sample of caregivers, some limitations should be noted. First, the data collected was predominantly from caregivers of White, non-Hispanic participants, who are relatively highly educated caregivers and from households with higher annual income, limiting the ability to generalize the findings to other demographic groups. These groups likely have overall better access to medical care, especially diagnostic evaluations. While efforts to increase the diversity of enrollment of underrepresented/marginalized groups involved patient advocacy group outreach, lacking was more robust methods to develop community partnerships to identify concerns and barriers to participation. Additionally, although participants were not required to receive clinical care at study sites and no clinical fees were associated with study participation, participants, and caregivers were required to travel to study sites and no compensation was provided to offset travel costs or lost wages. These issues represent potentially significant barriers to participation and likely contributed to the lack of participant diversity, and future work should recognize these issues, utilize more robust community engagement, provide resources to offset the financial burden associated with study participation, and incorporate methods such as remote assessment and online surveys to decrease the challenges related to the requirement to travel to study visits.

Second, most of the data available was from individuals with classic or atypical RTT with a smaller number of participants from other disorders (MDS, CDD, and FS). Conclusions from these groups should be tempered as a larger sample could give different results. While our experience with these disorders increases confidence in these results, further work is needed to confirm these findings.

Third, this study primarily utilized cross-sectional data from the US NHS obtained at the baseline visit, hence, we do not present data on the stability or change of these concerns over time for individuals. Additionally, the evolution in diagnostic practices and clinical care for people with RTT may contribute to differences in caregiver perception of significant clinical concerns. Future work utilizing the US NHS data for longitudinal analysis of caregiver concerns will provide an opportunity to address this limitation.

Fourth, caregivers were required to choose their top 3 concerns (and not more) and were required to uniquely rank choices (ties or equivalence was not allowed), limiting our knowledge of the depth of any one family’s concerns. However, the consistency of the rankings in this large cohort and the alignment of these concerns with clinical understanding of major features in RTT provide support that the findings accurately reflect the caregiver’s impression. While the pre-specified concern term list presented to caregivers was developed through literature review, expert clinical input, and RTT patient advocacy and caregiver input, it is possible that the range of items within the pre-specified concern term list did not completely represent the range of possible concerns. To address this, caregivers were provided the opportunity to select “other” and enter a free text response. While the free text response provided some additional information on concerns, overall “other” was infrequently selected and the concerns created from the free text were generally not high-frequency concerns. Fifth, the data is based on the caregiver’s concerns, rather than the affected individuals themselves. While capturing this information directly from affected individuals is optimal, the severe communication impairment in these disorders limits the ability for direct input from affected individuals.

Lastly, the study did not assess caregiver impressions on the relative impact of a specific concern nor the magnitude of change within a concern that would be meaningful. This represents an important avenue of future investigation that would further support the development and optimization of outcome measures for clinical trials in RTT and related disorders.

## Conclusion

The top concerns for individuals with RTT and RTT-related disorders are very similar across these different entities and are modified by age, clinical severity, and mutations as well as the specific diagnostic entity. The recognition of these caregiver concerns is critical in the development and selection of outcome measures for clinical trials, as instruments should either measure multiple domains simultaneously or a trial should incorporate multiple outcome measures to ensure assessment of top concerning features. This work provides foundational data on caregiver concerns for RTT and related disorders that should guide outcome measure development. Further, this study is aligned with FDA guidance [29, 30], including using caregiver information in lieu of patient concerns for those individuals with significant cognitive impairment [30], even though caregivers are likely to rate their impressions based on the symptoms they personally find most concerning. Failure to account for caregiver perceptions in these neurodevelopmental disorders may be viewed as a significant shortcoming by those responsible for providing care for these individuals. Consideration of caregiver concerns and caregiver impression of meaningful change deserves increased attention when assessing outcomes in future trials.

### Supplementary Information

**Additional file 1: Table S1. **MECP2 mutation distribution for Classic and Atypical RTT.

**Additional file 2: Table S2. **Prespecific concern choices and reclassified free text responses reclassified.

## Data Availability

The datasets from the Rett syndrome and Rett-related Disorders Natural History Study (NHS) have been deposited to the database of Genotypes and Phenotypes (dbGAP) repository, phs000574.v1.p1 and hyperlink to dataset(s) in https://www.ncbi.nlm.nih.gov/projects/gap/cgi-bin/study.cgi?study_id=phs000574.v1.p1, and are deposited to dbGAP per a predefined schedule at regular intervals. Additionally, datasets used for the analysis conducted within this work are available from the corresponding author on reasonable request and pursuant to any required data transfer and use agreements.
