# Supplementary material for: Top caregiver concerns in Rett syndrome and related disorders: data from the US natural history study
Source: J Neurodev Disord. 2023 Oct 13;15:33. doi: 10.1186/s11689-023-09502-z (PMC10571464; doi:10.1186/s11689-023-09502-z)
Supplement: Supplementary file 1 — Additional file 1: Table S1. MECP2 mutation distribution for Classic and Atypical RTT. [file 11689_2023_9502_MOESM1_ESM.pdf]

Additional file 1: Table S1: *MECP2* mutation distribution for Classic and Atypical RTT.

***MECP2* Mutation**	**Classic**	**Atypical**
Exon1	6	0
R106W	27	1
R133C	48	11
T158M	63	2
R168X	68	1
R255X	53	4
R270X	37	5
R294X	42	3
R306C	61	4
CTT	60	21
earlytrunc	48	7
LargeDel	52	4
Splice	10	1
OtherPt	46	8
MISSING	9	3
None	11	9
Total	641	84

Common recurrent point mutations in *MECP2* are identified by the specific mutation. Exon 1 includes all point mutations in exon 1. CTT is Carboxy-terminal truncations caused by a variety of insertions/deletions resulting in frameshift mutations. Early truncating mutations (earlytrunc) are a collection of nonsense or frameshift mutations before R294. Large deletions (LargeDel) are deletions that remove the majority of the *MECP2* coding sequence. Other point mutations (OtherPt) are all other missense or nonsense mutations in *MECP2*. None represents those individuals who had complete genetic testing for *MECP2* mutations, but no mutation was identified. Missing represents those participants in which no genetic mutation information was provided in the database.
