# Supplementary material for: Top caregiver concerns in Rett syndrome and related disorders: data from the US natural history study
Source: J Neurodev Disord. 2023 Oct 13;15:33. doi: 10.1186/s11689-023-09502-z (PMC10571464; doi:10.1186/s11689-023-09502-z)
Supplement: Supplementary file 2 — Additional file 2: Table S2. Prespecific concern choices and reclassified free text responses reclassified. [file 11689_2023_9502_MOESM2_ESM.pdf]

Prespecifiee concern choices and reclassified free text responses reclassified
**Abnormal Movements (other than hand stereotypies)**<br>abnormal myoclonic episodes<br>foot drop<br>freeze spells<br>lip smacking<br>motoric episodes<br>Movement Disorder<br>movement disorder (tremors and shaking episodes)<br>Rett Spells/Episodes<br>staring episodes<br>Temors/shaking<br>Twitching/shaking<br>uncontrolled movements
**Abnormal Walking/Balance Issues**<br>Abnormal Walking<br>absence of gait<br>Apraxia<br>Ataxia<br>Balance issues/unseteadiness while walking<br>Inability/cannot walk<br>gross motor skills/movement<br>Immobility<br>Lack of mobility<br>lack of movement/interaction<br>Lack of movement/motor skills<br>Limited motoric<br>loss of gross motor skills<br>Mobility<br>Mobility issues<br>Motor planning<br>Motor skills<br>no muscle movement (on ventilator/trach)<br>no walking/motor problems<br>Not able to ambulateusing wheelchair all the time<br>Not being able to bear weight or stand for transfers.<br>Not being able to perform AADLs on own<br>not sitting up<br>Not walking<br>sitting unsupported<br>transferring or moving her<br>Unable to sit<br>Unable to walk<br>Walking

**Air swallowing/Bloating/Excessive Gas**<br>excessive gas
**Constipation**<br>Can't control bowel movements<br>constipation/relux<br>Difficulty/refusal of drinking, leading to constipation<br>GI issues- coordinating pooping<br>GI issues; gastroparesis, Superior Mesyntaric Artery Syndrome, constipation, GERD<br>GI issues: constipation and reflux
**Frequent infections**<br>Frequent illness<br>frequent infections<br>Infections<br>Lunch infections secondary to viruses and to aspiration<br>multiple viruses, aspiration pnuemonias<br>Pneumonia<br>recurrent hospitalizations<br>Respiratory infection<br>Sick easily<br>Skin infection<br>Skin infections<br>strep infection of the throat
**Gastroesophageal reflux**<br>GI issues, possible reflux<br>reflux
**Lack of effective chewing or swallowing**<br>aspiration<br>Chew<br>chewing/swallowing<br>choking<br>choking on secretions<br>feeding<br>feeding difficulty<br>Issues with eating, lack of interest/issues eating<br>Lack of chewing, swallowing<br>Not being able to eat<br>NPO-Tube fed<br>NPO/no food by mouth<br>Poor stamina for chewing swallowing

swallowing
Tube feeding

Lack of effective communication
Communication
Consistent/effective communication
hyper vocalizations
lack of speech or sounds
non verbal
non-verbal
reading
repetitive nonsensical speech pattern
repetitive speech
spelling

Lack of hand use
difficulty feeding self
feeding herself
Fine Motor

Problems with sleep
awake/ sleep cycles (predominantly 20ish hrs a day)
central sleep apnea
Excessively sleepy
getting enough continuous sleep, staying asleep>6hrs
Problems with sleep while traveling
sleep
sleep apnea
sleep, staying asleep
snoring

Rapid breathing or breath holding while awake
breathing movements
Hyperventilation
stops breathing awake OR asleep

Repetitive hand movements (wringing, mouthing)
everything gets put in her mouth
Hand stereotypies
hand to mouth
Mouthing
near constant self stimulation including vocalizations and movements

**Scoliosis**<br>Kyphosis<br>kyphosis of 100 degrees<br>scoliosis
**Screaming episodes**<br>Being tired, which causes screaming episodes
**Seizures**<br>Possible seizures
**Self-abusive behaviors**<br>Always picking at scabs/wounds<br>screaming with self abusive behaviors simultaneous

Created concerns and reclassified free text responses reclassified
**Attention/Cognition/Developmental Delay/ID**<br>ADHD symptoms<br>Attention<br>attention deficit disorder<br>behavior/attentiveness<br>cognition<br>Cognitive<br>cognitive ability<br>Cognitive declone or confusion outside of routine<br>cognitive delay<br>cognitive functioning<br>Cognitive impairment<br>developmental delay<br>Developmental/Intellectual delay<br>General developmental delays<br>general disinterest in toys and the world around him<br>inability to interact appropriately with the world around him<br>Learning Ability<br>learning delays<br>math<br>memory<br>visual and auditory orientation towards stimuli
**Drooling/Spitting**<br>Drooling<br>lots of mucus in the afternoons<br>Raspberries<br>spit up<br>Spitting
**Dystonia/Rigidity/Contractures**<br>abnormal tone<br>Contracture<br>contractures, muscle weakness/tightness<br>contractures/increased tone<br>Dystonia<br>Excessive tone<br>high muscle tone (dystonia)<br>hypertonia<br>increased tone<br>More stiff<br>Muscle spasticity<br>Muscle Stiffness

muscle tightness<br>muscle tone<br>Rigidty<br>Spasticity<br>stiffening<br>Stiffness<br>Stiffness and rigidity<br>tight muscles in legs
**fatigue/lethargy/energy**<br>Episodes of fatigue<br>Fatigue<br>lethargic<br>lethargy<br>Low stamina causing less participations to activities<br>strength<br>tiredness
**GU issues**<br>incontinence<br>incontinent<br>need for revision g-tube surgery and kidney stones<br>neurogenic bowel and bladdar<br>renal problems<br>Urinary retention<br>urine and bowel retention<br>Urine retention
**Hypotonia**<br>head control<br>head control, low muscle tone<br>Hypotonia<br>low muscle tone<br>low tone
**None indicated**<br>n/a<br>N/A- caregiver could not identify a 3rd problem.<br>None<br>None indicated<br>Not applicable<br>nothing

Other Autonomic
Autonomic storms
heart issues SVT (supraventricular tachycardia)
Hypothermia

Other Behavior
ability to interact with peers
agitation
autism-like symptoms
behavior issues
behavior when she doesn't get her way
behaviors and moods during menstrual cycle
complete lack of response to any stimuli other than pain
Emotional
emotional: unsure why. could be pain, anxiety, depression or puberty
Fits
General safety concerns: she would walk into the street if not watched.
irritability
lack of affect to show mood/emotion
lack of awareness of personal safety (falling, bolting)
Lack of independence
lack of toilet training
maladaptive behaviors
Mood Swings
moody, crying spells, seems upset
need for attention
no interest in toilet-training
not being able to be "normal"
OCD
pica
Potty training
problems with change
Safety
self care independence
sensory issues
Sensory processing
social interaction
toilet training
toileting
Using bathroom

Other GI
abdominal pain
Bowel dysmotility

bowels<br>Colitis<br>cramping<br>desire to eat<br>eating<br>Eosinophilic esophagitis<br>excessive weight<br>excessive weight gain<br>food allergies<br>G-tube<br>gall bladder - no disgnosed disorder<br>gall bladder issues<br>gallbladder sludge<br>Gastrointestinal issue<br>gastroporesis<br>GI<br>GI concerns- reduced motility<br>GI issues<br>GI issues - Stomach not emptying<br>GI problems; loose stool<br>GI tract<br>GI Upset<br>growth<br>loose bowls<br>nutrition<br>Over Eating<br>Overall weight<br>Overweight<br>pain/discomfort from gtube<br>picky eater<br>rapid weight gain/weight maintenance<br>Stomach pain<br>Vomiting<br>Weight gain<br>weight gain/eating difficulty
**Other Health Issue**<br>Anemia<br>Blisters on feet/ingrown toe nail infection<br>bone density<br>bones are weak<br>Colon Cancer<br>coughing fits<br>COVID 19 routine disruptions

Dental Issues<br>difficulties with equipment<br>Eczema<br>hearing loss<br>Low Ferritin<br>nose congestion<br>physical impairment<br>side effects/med management
**Other musculoskeletal**<br>hip dysplasia<br>hip out of socket<br>Hips dysplasia<br>muscle loss<br>muscle spasms
**pain issues**<br>headaches<br>Joint pain<br>pain<br>pain, health issues
**Respiratory/pulmonary**<br>Airway problems (due to tracheotomy)<br>mucus production/blocking airway<br>On oxygen at night now<br>Respiratory<br>respiratory acidosis<br>Respiratory issues
**Therapy issues**<br>Lack of therapy<br>Loss of skills not in practice
